# Characterization and discrimination of flavor and taste profiles in olive oil from different varieties using E-nose, GC-IMS, E-tongue, and UPLC-MS/MS

**DOI:** 10.1016/j.fochx.2026.104354

**Published:** 2026-08-25

**Authors:** Boxiao Wu, Churan Li, Qing Hu, Bin Lu, Changwei Cao, Fang Li, Huan Kan, Yun Liu

**Affiliations:** a Forest Resources Exploitation and Utilization Engineering Research Center for Grand Health of Yunnan Provincial Universities, Southwest Forestry University, Kunming 650224, China; b Yunnan Technology Innovation Center of Woody Oil, Yunnan Academy of Forestry and Grassland, Kunming 650204, China

**Keywords:** Olive oil, Flavor, Taste, GC-IMS, UPLC-MS/MS, Molecular docking

## Abstract

The varieties of *Olea europaea* play an important part in forming flavor and taste profiles of olive oil. Herein, extra virgin olive oil (EVOO) of five *O. europaea* varieties were comprehensively characterized by flavor and taste using an electronic nose (E-nose), gas chromatography-ion mobility spectrometry (GC-IMS), electronic tongue (E-tongue), and ultra performance liquid chromatography tandem mass spectrometry (UPLC-MS/MS). E-nose effectively differentiated the samples. Thirty volatile organic compounds were characterized, with esters being the most abundant. E-tongue profiling indicated that all five EVOO exhibited sweetness, bitterness, and astringency, albeit with differentiated intensities. Comparative analyses and Mantel test analyses identified 26 phenolic compounds underlying contributors to bitterness and astringency in the EVOO. Molecular docking approaches were employed to validate and visualize the capabilities for five bitter compounds with high affinity combining to bitter taste receptors. This research revealed the metabolic regulation responsible for flavor and taste profiles of EVOO.

## Highlights

• The flavor and taste of five extra virgin olive oils (EVOOs) were characterized.
• ROAV analysis revealed 16 VOCs that contribute to the flavor profile of EVOOs.
• UPLC-MS/MS was employed to examine composition of phenolic compounds in five EVOOs.
• Twenty-six phenolic compounds caused the bitterness and astringency in EVOOs.

## 1 Introduction

*Olea europaea* L., native to the Mediterranean region, is an economically important woody oil crop with a cultivation history of more than 4000 years (Spangenberg & Lantos, 2024). In the 1960s, *O. europaea* was introduced into China and cultivated extensively, primarily in Gansu, Sichuan, and Yunnan. Lijiang City in Yunnan Province became one of the main cultivation areas because of its similarities with the Mediterranean climate (Su et al., 2018). As the main processing product derived from the fruit of *O. europaea*, olive oil exhibits extremely high values in dietary and medicinal applications and has been designated “liquid gold” and “the queen of vegetable oils” (Ortiz-Arrabal et al., 2023). Olive oil is characterized by a fatty acid profile consisting of comparatively higher proportions of unsaturated fatty acids and lower proportions of saturated fatty acids (Spangenberg & Lantos, 2024). Compared with other vegetable oils, olive oil contains abundant unsaponifiable components like phytosterols, tocopherols, and squalene, as well as phenolic components, including hydroxytyrosol, oleuropein, and oleocanthal. These components confer health advantages like antioxidant, anti-inflammatory, anti-analgesic, anti-viral, and anti-aging effects (Frankel, 2011). With the improvement of lifestyle and health awareness, consumer demand and industrial production of olive oil have grown rapidly in China.

Besides health benefits, flavor and taste profiles are important factors influencing food selection (Danna et al., 2021). Food flavor chemistry primarily focuses on the composition, sources, and formation mechanisms of flavor compounds in food. These insights guide the optimization of food processing techniques, thereby enhancing product sensory quality, controlling flavor stability, and facilitating the development of novel flavor products (Sung et al., 2025). In recent years, electronic nose (E-nose), gas chromatography-ion mobility spectrometry (GC-IMS), electronic tongue (E-tongue), ultra performance liquid chromatography tandem mass spectrometry (UPLC-MS/MS), and relative odor activity value (ROAV) have been used to evaluate food flavor and taste (Liu et al., 2024; Wang, Ma, et al., 2025). The sensory attributes of olive oil encompass flavor and taste. The extra virgin olive oil (EVOO) exhibits positive flavors that are described as fruity and grassy, whereas inferior olive oils exude mal odors such as musty and rancid (Kalua et al., 2007). The flavor components in olive oil primarily consist of C_6_ aldehydes, alcohols, ketones, and esters, which are formed through the lipoxygenase pathway and are significantly influenced by *O. europaea* varieties (Cecchi et al., 2022). The taste in olive oil results from phenolic compounds and generally manifests as bitterness, astringency, and pungency (Harzalli et al., 2018). International Olive Council and the European Union have established standards for olive oil sensory attributes to identify their its attributes and defects in flavor (Caño-Carrillo et al., 2024). However, traditional sensory assessment patterns for olive oil are characterized by individual differences and subjectivity resulting from physiological differences, experiential biases and cultural preferences. To overcome these limitations, modern instrumental analysis has emerged as an indispensable tool in EVOO research (Alahlah et al., 2025). Rodrigues et al. (2019) found that a single E-tongue measurement could simultaneously predict various physicochemical parameters and sensory attributes of olive oil, underscoring its great promise as an industrial-grade tool for rapid and comprehensive quality assessment. Damiani et al. (2020) confirmed the reliability and effectiveness of GC-IMS and E-nose in grading olive oil quality, highlighting their potential as rapid, high-throughput, and non-destructive analytical tools.

In this study, the flavor profiles of EVOO from five *O. europaea* varieties were evaluated using E-nose, GC-IMS, E-tongue, and UPLC-MS/MS. Food flavor chemistry was conducted for identifying phenolic compounds in five EVOOs, and comparative analysis approaches were utilized to reveal the underlying phenolic compounds contributing to bitterness and astringency. The findings could offer scientific bases toward cultivating *O. europaea* varieties with an excellent flavor profile and developing high-quality EVOO products.

## 2 Materials and methods

### 2.1 Sample treatment

The five varieties of single-cultivar fruits of *O. europaea* collected in October 2023 from Yongsheng County, Lijiang City, Yunnan Province, China, were free from pest infestation and physical damage. All olive samples of consistent maturity were harvested at turning-color stage, determined using the olive maturity index measurement method (Bengana et al., 2013). All fruits were processed within 24 h after harvest. Specifically, all five olive fruits were washed, crushed using a hammer crusher, malaxated at 30 °C for 40 min, and centrifuged to obtain five single samples of olive oil. All five olive oils met the EVOO requirements of International Olive Oil Council, Codex Alimentarius Commission and GB/T 23347–2021. They were designated Arbequina (AQ), Ezhi-8 (EP), Picual (PC), Frantoio (FO), and Koroneiki (KK), and stored in dark until for further analysis.

Moreover, 20 g EVOO was added to 50 mL of 20% ethanol, homogenized for 30 s, vortexed for 3 min, and ultrasonicated for 15 min. Mixtures were then centrifuged at 5000 rpm for 25 min under 4 °C, and filtrated over 0.22 μm filters for E-tongue and UPLC-MS/MS analyses.

### 2.2 E-nose analysis

Flavors of five EVOOs were evaluated by E-nose (PEN 3, AIRSENSE, Germany) outfitted with ten transducers (Table S1). Specifically, 2.00 g EVOO was precisely placed in headspace bottles and equilibrated under 30 °C for 20 min before sample injection. Carrier gas consisted of pure air flowing at 400 mL/min. Sampling interval, sensor washing, and measurement times were 5, 90, and 120 s, respectively.

### 2.3 GC-IMS analysis

Volatile organic compounds (VOCs) in five EVOOs were identified by GC-IMS (Flavor Spec1H1–00053, GAS, Germany) equipped with an MXT-5 column (MXT-5, 15 m × 0.53 mm × 1 μm, RESTEK, USA). Specifically, 2.00 g EVOO was subjected to incubation in headspace bottles at 60 °C and at 500 rpm for 15 min before injection. Drift gas consisted of nitrogen flowing at 400 mL/min. Carrier gas consisted of nitrogen with an initial flow rate of 2.0 mL/min for 2 min, followed by an increase to 10 mL/min for 8 min, then gradually increased to 100 mL/min for 10 min, and maintained at 100 mL/min for 10 min. Injection needle and IMS were set for 85 and 45 °C respectively. VOCs data visualization and analysis were performed through the Laboratory Analytical Viewer, and VOCs were identified based on retention indices, drift indices, and the NIST library.

### 2.4 ROAV analysis

ROAV analysis was conducted according to the method of Wang, Ma, et al. (2025). The flavor profile was identified using the Perflavory Information System (http://www.perflavory.com/index.html). The formula for calculating ROAV was as follows:

(1) $$\text{ROAV}=\frac{C_{i}}{C_{\text{stan}}}\times \frac{T_{\text{stan}}}{T_{i}}\times 100$$

where *C*_*i*_ (%) and *T*_*i*_ (μg/kg) denote relative content and odor threshold of VOCs in EVOO, respectively, and *C*_*stan*_ (%) and *T*_*stan*_ (μg/kg) represent relative content and odor threshold of the most abundant VOCs in EVOO, respectively.

### 2.5 E-tongue analysis

Taste of five EVOOs was assessed by E-tongue (SA402B, Insent, Japan) outfitted with six transducers. The types and corresponding taste signals are listed in Table S2. Specifically, 40 mL EVOO ethanol extract was added to headspace bottles, and electrochemical scanning was performed using E-tongue sensors. The sensor was equilibrated with wash and reference solutions, and data collection time was 40 s with collection period of 1 s. Each sample was analyzed in quadruplicate, with final test results averaged from the last three replicates.

### 2.6 Food flavor compound analysis

The EVOO ethanol extract samples were characterized using UPLC-MS/MS (ExionLC™ AD, SCIEX, USA) which was equipped with a 2.1 mm × 100 mm × 1.8 μm column (Agilent SB-C18, Agilent, USA). Eluents A and B were ultrapure water containing 0.1% formic acid and acetonitrile containing 0.1% formic acid, respectively. An elution program was performed at 0.00–9.00 min, 95%–5% A; 9.00–10.00 min, 5% A; 10.00–11.10 min, 5%–95% A; 11.10–14.00 min, 95% A. The injection volume, column temperature, and flow rate were 2.0 μL, 40 °C, and 0.35 mL/min, respectively. The operating voltages for MS were 5500 V and − 4500 V in the positive and negative ion modes, respectively. The ion source gas I, gas II, and curtain gas pressures were set at 50, 60, and 25 psi, respectively. Scanning mode was multiple reaction monitoring mode over the range of 50–1000 *m/z*. Phenolic metabolites were recognized by matching MS and MS/MS data with Metware self-built database (Metware Biotechnology Co., Ltd., Wuhan, Hubei, China). Quality control was established by mixing sample extracts and inserting them into each tenth sample to evaluate repeatability and reliability for analytical procedures.

### 2.7 Molecular docking

Molecular docking was conducted as described by Shi, Xiao, et al. (2025) to identify the bitterness between phenolic compounds and bitter taste receptor proteins. The 3D structure files for the active compounds in an SDF format were obtained from PubChem database (https://pubchem.ncbi.nlm.nih.gov/) and converted to the Protein Data Bank format with the OpenBabel program. Bitter taste receptor protein structures of TAS2R14 (UniProtKB ID: Q9NYV8) and TAS2R39 (UniProtKB ID: P59534) were retrieved from UniProt (https://www.uniprot.org/). The proteins were dehydrated and hydrogenated, then semi-rigid docked with hydrogenated compounds and combining energies were determined with AutoDockTools-1.5.7 tool. The docking results were visualized using PyMol (DeLano Scientific LLC, CA, USA) and Discovery Studio software (BIOVIA, Paris, France).

### 2.8 Statistical analyses

Data were analyzed with SPSS Statistics 27 software (IBM, NY, USA) with *p* < 0.05 being statistically significance. The results were presented by the mean ± standard deviation. The figures were drawn using Origin 2024b (OriginLab, MA, USA) and GraphPad Prism 10 (GraphPad Software, CA, USA). The Orthogonal Partial Least Squares Discriminant Analysis (OPLS-DA) was performed using Simca 14.1 (MKS Umetrics, Malmö, Sweden).

## 3 Results and discussion

### 3.1 E-nose analysis

**Fig. 1:**
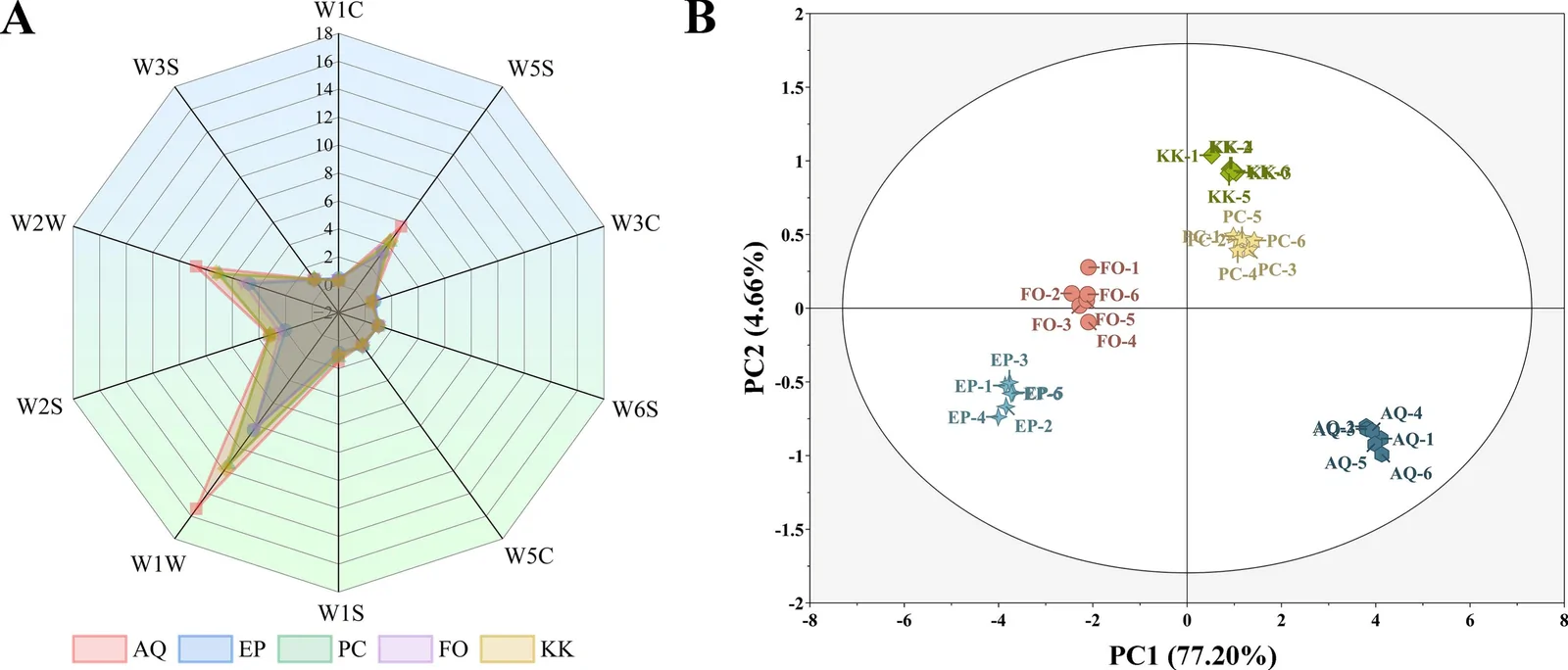
E-nose analysis of five EVOOs. (A) Radar chart; (B) PCA.

E-nose is an odor detection apparatus that simulates olfactory system through sensor arrays. It enables rapid multi component identification based on the differential responses of various flavor molecules, which has been widely used in the food analysis field (Chen, Yang, et al., 2024; Kanwal & Musharraf, 2024). The overall flavor radar charts of the five EVOOs detected by E-nose are shown in Fig. 1A. The ten sensor responses to the five EVOOs were similar in shape, which suggested similar overall flavor profiles. Notably, the response values for W1W, W2W, and W5S, which are sensitive to inorganic sulfides, aromatic components and organic sulfides, and nitrogen oxides, respectively, were remarkably higher than other sensors (*p* < 0.05). These are consistent with finding of Lü et al. (2021). The total response values of ten sensors to five EVOOs also differed significantly, with AQ exhibiting remarkably higher values compared to other four EVOOs, indicating more intense flavor characteristics in AQ (Zhang et al., 2025). Fig. 1B showed that total explained variance for the principal component analysis (PCA) from the five EVOOs for the E-nose was 81.86%, of which 77.20% was for PC1 and 4.66% for PC2. This indicates that the data were effective at capturing the combined information for the whole sample (Moreira et al., 2022). The various colored zones denote the overall volatiles distribution across the five EVOOs, with the distance between zones represents differences between samples (Ongalbek et al., 2024). KK and PC were located in the same quadrant, while FO, EP, and AQ were positioned in different quadrants, which indicated that the differences between KK and PC were relatively lower, whereas the differences among the other groups were more pronounced (Fig. 1B).

### 3.2 GC-IMS analysis

**Fig. 2:**
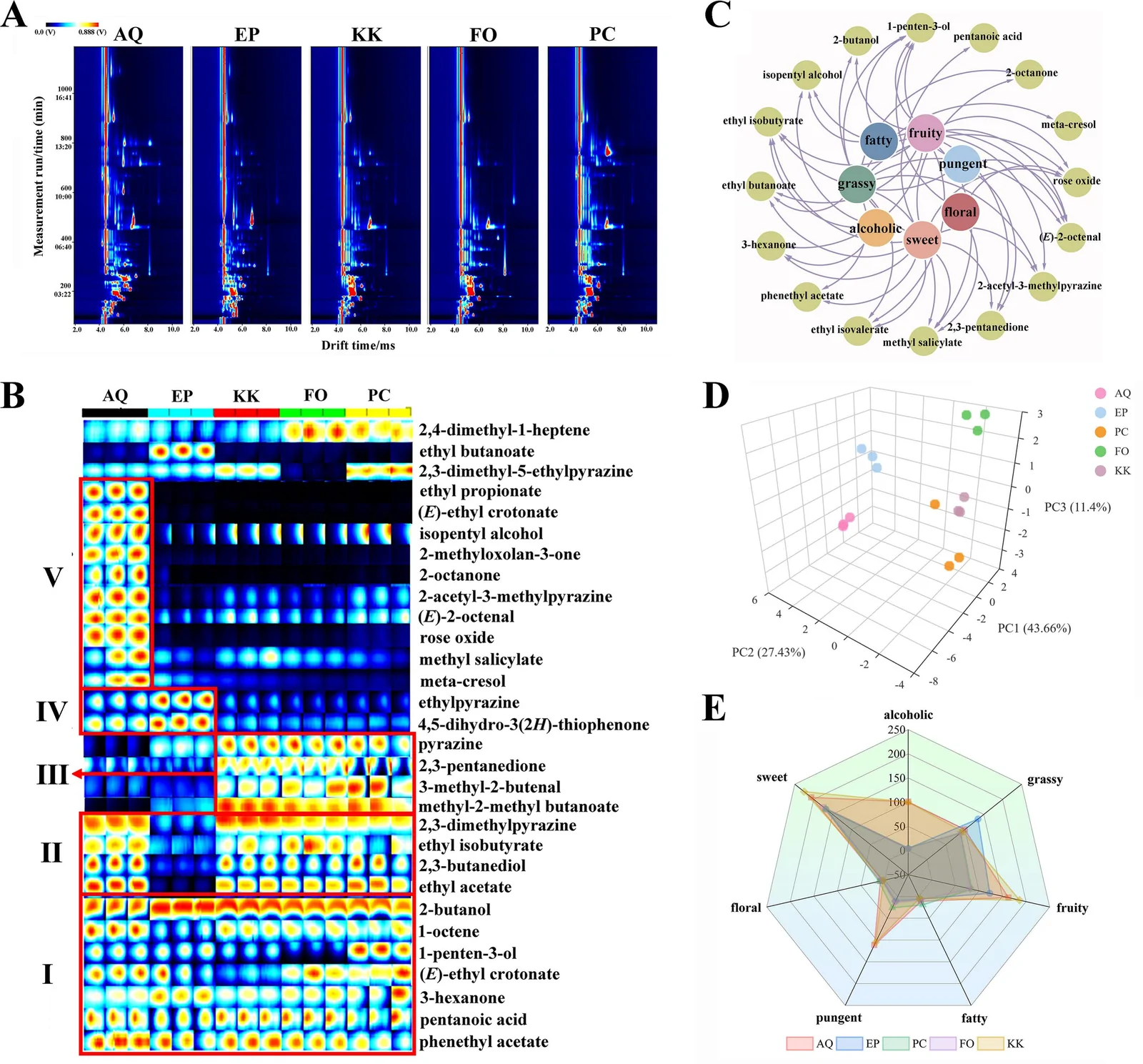
Characterization and analysis of VOCs in five EVOOs by GC-IMS. (A) top view of GC-IMS; (B) VOC fingerprints; (C) VOC odor description; (D) PCA of VOCs; (E) flavor profile of five EVOOs.

Differences in VOCs among the five EVOOs were determined using GC-IMS. The 3D-topographic maps and top view for five EVOOs are shown in Fig. 2A, where the points in right side of red active ion peaks denote VOCs, with depth of color representing their concentration (Xie et al., 2025). The results indicated that the five EVOOs contained similar VOC types, with clear differences in their contents.

Thirty VOCs were recognized in five EVOOs, which included 8 esters, 7 aromatic hydrocarbons, 5 ketones, 4 alcohols, 2 aldehydes, 2 olefins, 1 ether, and 1 acid (Table S3). VOC fingerprint profiles of five EVOOs are shown in Fig. 2B, where each point corresponds to a VOC, and a redder color indicates a higher compound concentration (Shi et al., 2025). Phenethyl acetate, pentanoic acid, 3-hexanone, (*E*)-ethyl crotonate, 1-penten-3-ol, 1-octene, and 2-butanol were detected in all five EVOOs at high concentrations (Fig. 2B, Section I). These compounds primarily contributed fruity and grassy flavors (Fig. 2C). Ethyl acetate, 2,3-butanediol, ethyl isovalerate, and 2,3-dimethylpyrazine were detected in AQ, KK, FO, and PC (Fig. 2B, Section II), contributing fruity and sweet flavors (Fig. 2C). Methyl-2-methyl butanoate, 3-methyl-2-butenal, 2,3-pentanedione, and pyrazine were distributed across KK, FO, and PC (Fig. 2B, Section III), with 2,3-pentanedione and pyrazine exhibiting fruity, pungent, and sweet flavors (Fig. 2C). Notably, 4,5-dihydro-3(2*H*)-thiophenone and ethylpyrazine were typical VOCs in AQ and EP, yet did not contribute to the overall flavor profile (Fig. 2B, Section IV). Moreover, meta-cresol, methyl salicylate, rose oxide, (*E*)-2-octenal, 2-acetyl-3-methylpyrazine, 2-octanone, 2-methyloxolan-3-one, isopentyl alcohol, (*E*)-ethyl crotonate, and ethyl propionate were unique to AQ (Fig. 2B, Section V), imparting fruity, grassy, and sweet flavors (Fig. 2C).

The Fig. 2D showed the PCA analysis for the five EVOOs, where PC1, PC2, and PC3 explained 43.66%, 27.43%, and 11.40% of the variance, respectively. This indicated that the principal components collectively captured the majority information within the samples. Moreover, the five EVOOs were clearly distinguished in Fig. 2D, consistent with findings of E-nose analysis (Fig. 1B).

### 3.3 ROAV analysis

Flavors contributed by VOCs depends on their content and odor threshold, which is frequently represented by ROAV, the higher the ROAV, the greater the contribution of VOC to the total flavors (Ping et al., 2024). Specifically, the VOCs with ROAV >1 are usually considered the main contributors toward the total flavors, whereas VOCs with 0.1 ≤ ROAV ≤1 are considered important flavor modifiers (Wang, Ma, et al., 2025).

To identify the VOCs that significantly influence the overall flavor profiles of the five EVOOs, ROAVs were calculated based on their relative contents. As listed in Table 1, esters contributed most importantly to overall flavors in five EVOOs, probably due to their lower odor thresholds and distinctive flavor characteristics (Li et al., 2025). In addition, ethyl isobutyrate contributed a significant effect on the flavors of AQ and KK, which primarily exhibited fruity, pungent, alcoholic, and fatty flavors. Ethyl butanoate manifested the strongest influence on EP with fruity, grassy, and sweet flavors. Pentanoic acid showed the most pronounced effect on PC and FO, primarily exhibiting sweet flavors.

**Table 1:** ROAV of key VOCs in five EVOOs.

Classification	VOCs	Odor threshold (μg/g)	Odor description	ROAV				
				AQ	EP	PC	FO	KK
Esters (4)	ethyl isobutyrate	0.00011	fruity, pungent, alcoholic, fatty	100.00	4.06	4.65	3.98	100.00
	ethyl butanoate	0.00005	fruity, grassy, sweet	18.88	100.00	17.69	21.52	62.52
	ethyl isovalerate	0.00007	fruity, grassy, sweet	25.25	10.73	36.88	42.13	16.25
	phenethyl acetate	0.01600	fruity, sweet	1.46	1.10	2.04	2.17	1.11
Aromatic hydrocarbon (3)	meta-cresol	0.00044	grassy	37.77	16.17	10.85	11.77	8.19
	methyl salicylate	0.00670	fruity, grassy, floral, sweet	2.85	1.09	1.88	2.66	1.56
	2-acetyl-3-methylpyrazine	0.02000	fruity, grassy, sweet	0.46	0.06	0.40	0.16	0.13
Alcohols (3)	isopentyl alcohol	0.00610	fruity, alcoholic, fatty	0.79	0.17	0.85	0.54	0.15
	1-penten-3-ol	0.01000	grassy, pungent, alcoholic, sweet, floral	3.43	3.03	9.98	2.45	0.92
	2-butanol	0.13000	fruity, sweet	0.30	0.22	0.49	0.46	0.12
Ketones (3)	2-octanone	0.23000	fruity, sweet	0.11	0.01	0.01	0.01	–
	3-hexanone	0.04100	grassy, alcoholic, sweet	0.05	0.05	0.13	0.10	0.03
	2,3-pentanedione	0.01000	fruity, pungent, sweet	1.05	1.15	2.84	2.59	1.36
Acids (1)	pentanoic acid	0.00016	sweet	53.41	49.18	100.00	100.00	42.16
Aldehydes (1)	(*E*)-2-octenal	0.00270	fruity, grassy, pungent, fatty	6.94	2.45	8.58	5.01	3.14
Ethers (1)	rose oxide	0.00400	fruity, grassy, floral, sweet	2.66	0.24	0.54	0.41	0.19

Notably, the contribution of esters in five EVOOs was reflected not only in their high ROAV values but also in their cultivar distributions. Specifically, Ethyl isobutyrate was dominant in AQ and KK, ethyl butanoate was most prominent in EP, and ethyl isovalerate exhibited strong presence in PC and FO. These findings suggested that the differentiation of EVOO flavor profiles was not simply determined by the total abundance of esters, but rather by the specific distribution of individual ester compounds. Studies have found that olive varieties significantly influence the formation of VOCs associated with sensory characteristics like fruity, sweet, and grassy flavors, and that esters may serve as key contributors to cultivar-dependent odor differences in EVOOs (Cecchi et al., 2021; Tomé-Rodríguez et al., 2021). Additionally, Ríos-Reina et al. (2023) also demonstrated that esters were closely related to grassy and fruity sensory attributes in EVOOs, further supporting the role of esters in shaping differentiated sensory profiles.

As shown in Fig. 2E, the overall flavor profiles of the five EVOOs comprised alcoholic, grassy, fruity, sweet, fatty, floral, and pungent flavors, with sweet, grassy, and fruity flavors predominating. The overall flavor profiles of EVOOs are likely shaped by the combined contribution of multiple odor-active compounds rather than by a single dominant VOC (Chen et al., 2025). Herein, several compounds with high ROAVs shared overlapping sensory descriptors, suggesting that they may jointly contribute to the perceived flavor attributes of different EVOO varieties. Specifically, Ethyl isobutyrate, ethyl butanoate, ethyl isovalerate, and phenethyl acetate may collectively enhance fruity and sweet notes, whereas meta-cresol, 1-penten-3-ol, methyl salicylate, and (*E*)-2-octenal may contribute to grassy and pungent perceptions. These overlapping sensory attributes suggested that the flavor differences among the five EVOOs might arise from the balance and co-occurrence of key VOCs rather than from individual ROAV, which is consistent with the role of VOCs in the sensory characterization of virgin olive oils (Cecchi et al., 2021). Moreover, the release and perception of VOCs in EVOO might also be influenced by the oil matrix and minor components. Research has shown that phenols and saliva could affect the release of volatile compounds and contribute to the perception of odor in virgin olive oil (Díaz-Montaña et al., 2024). From the food-matrix perspective, interactions between odorants and non-volatile matrix components including lipids and polyphenols, could alter odorant volatility, thereby influencing odor perception (Wang, Wang, et al., 2025).

### 3.4 E-tongue analysis

Taste characteristics of five EVOOs were compared via E-tongue. Aftertaste-bitterness, aftertaste-astringency and richness reflect persistence of bitter, astringent and umami sensations, respectively (Chen, Yin, et al., 2024). Potential difference response values for nine taste sensations in the five EVOOs were calculated using tasteless as a reference. Table 2 and Fig. S1 indicated that remarkable differences were observed in response values among the five EVOOs, which facilitated the differentiation of the EVOOs from different varieties. Among the five EVOOs, the taste potential difference response values of sweetness were the highest, followed by bitterness and astringency. This indicates that sweetness, bitterness, and astringency primarily influence the taste profiles of the five EVOOs (Cai et al., 2024). This observation agreed to findings from Genovese et al. (2021). In addition, total explained variance of PCA for the five EVOOs using the E-tongue was 88.76%, of which 73.61% was for PC1 and 15.15% for PC2. This indicates that the data were effective at capturing the combined information of the whole sample efficiently. The spatial distribution of the five EVOOs tastes showed a clustering trend in PC1 and PC2, which can be roughly classified into two categories, in which KK was presented as a category alone, and AQ, FO, EP, and PC were classified into one category, as their spatial distributions were closer to one another. The spatial distance of KK was distributed further compared with the other four EVOOs, which indicates that KK is different from the other samples, whereas AQ, FO, EP, and PC are more consistent in terms of taste profile.

**Table 2:** Taste potential difference response values for E-tongue in five EVOOs.

Taste	AQ	EP	PC	FO	KK
sourness	−16.22 ± 0.70^b^	−15.72 ± 0.39^b^	−17.86 ± 0.44^c^	−19.49 ± 0.23^d^	−11.56 ± 0.12^a^
bitterness	13.43 ± 0.48^b^	13.10 ± 0.73^b^	12.92 ± 0.34^b^	12.52 ± 0.29^b^	16.25 ± 0.42^a^
astringency	2.49 ± 0.04^e^	3.68 ± 0.07^b^	3.22 ± 0.09^c^	2.93 ± 0.20^d^	4.20 ± 0.06^a^
aftertaste-bitterness	0.41 ± 0.20^c^	1.10 ± 0.34^b^	0.30 ± 0.24^c^	0.53 ± 0.12^c^	4.74 ± 0.50^a^
aftertaste-astringency	0.24 ± 0.01^b^	0.20 ± 0.05^b^	0.27 ± 0.27^b^	0.42 ± 0.05^b^	0.70 ± 0.18^a^
umami	0.09 ± 0.04^c^	0.36 ± 0.12^b^	−0.20 ± 0.20^d^	−0.23 ± 0.12^d^	0.99 ± 0.16^a^
richness	1.16 ± 0.05^a^	0.95 ± 0.05^b^	1.00 ± 0.02^b^	0.99 ± 0.07^b^	0.63 ± 0.06^c^
saltness	1.09 ± 0.17^bc^	1.53 ± 0.24^a^	0.97 ± 0.26^c^	1.02 ± 0.21^bc^	1.38 ± 0.10^ab^
sweetness	23.43 ± 0.70^a^	23.85 ± 0.25^a^	23.45 ± 0.42^a^	23.33 ± 0.07^a^	19.64 ± 1.78^b^

Significant differences between groups are indicated by different letters in the same row of data (*p* < 0.05).

### 3.5 Food flavor compound analysis

**Fig. 3:**
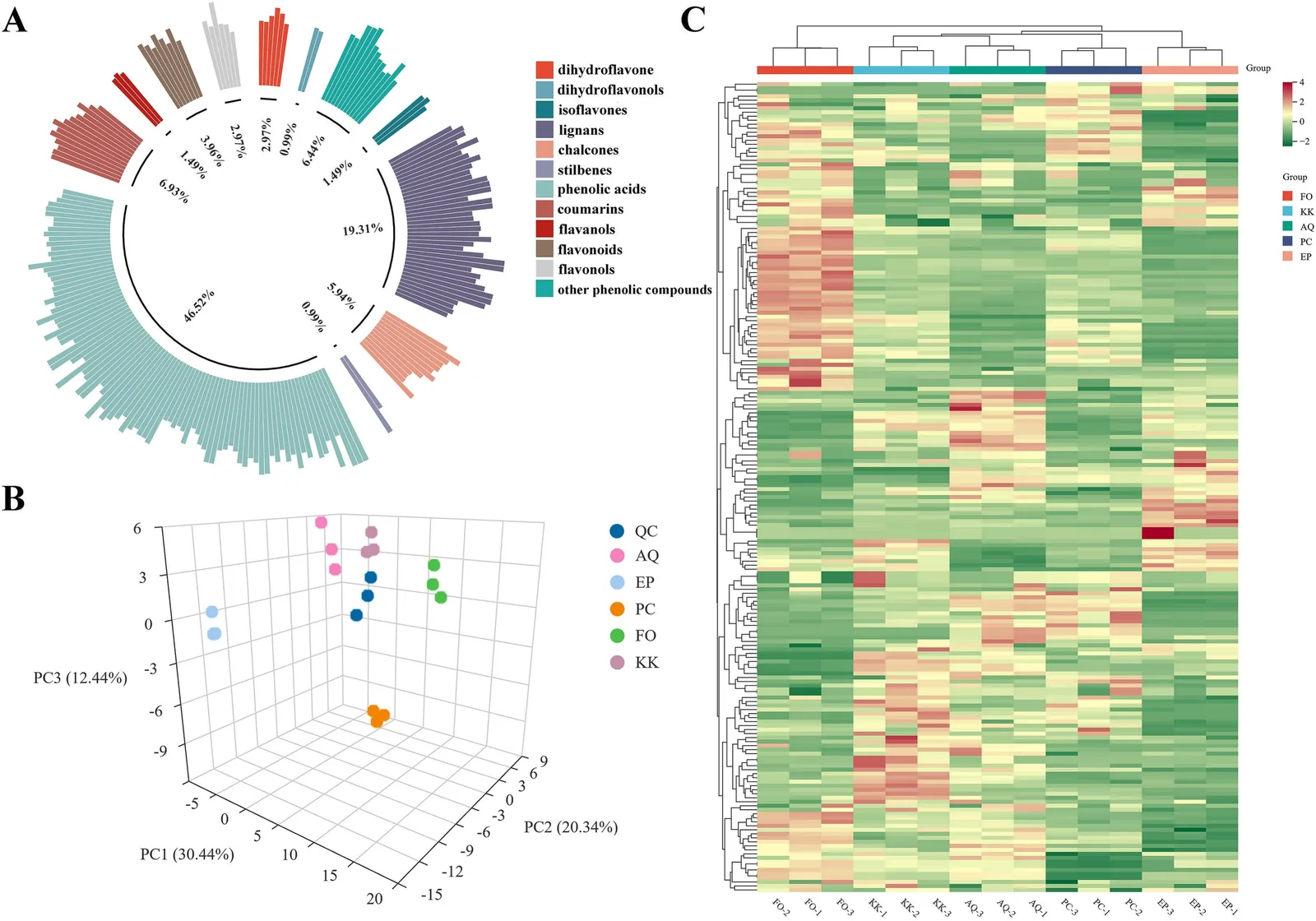
Analysis of phenolic metabolites in five EVOOs. (A) the distribution of phenolic metabolites; (B) PCA; (C) clustering heatmap.

Apart from biological activity, phenolic compounds are also important components that contribute to food taste (Zhang et al., 2024). Phenolic compound compositions in the five EVOOs were analyzed by UPLC-MS/MS, and 202 phenolic compounds were recognized (Fig. 3A). These metabolites were primarily classified into 12 groups, which included phenolic acids (94), lignans (39), coumarins (14), chalcones (12), flavonoids (8), dihydroflavonoids (6), flavonols (6), isoflavonoids (3), flavanols (3), dihydroflavonols (2), stilbenes (2), and other phenolic compounds (13). Among these, phenolic acids exhibited the maximum contents, consistent with the findings of Tang et al. (2018).

The differences in phenolic metabolites among the five EVOOs were analyzed by PCA (Fig. 3B). The contributions of PC1, PC2, and PC3 to the phenolic compounds of the five EVOOs were 30.44%, 20.34% and 12.44%, respectively, for a total of 63.22%. This accounted for most of the information in the five EVOOs. Moreover, the distribution of the five EVOOs was more dispersed, indicating that the phenolic compounds exhibited a large variation. Consequently, the five EVOOs could be distinguished according to their phenolic profiles.

Phenolic metabolites among the five EVOOs were visualized using clustering heatmaps. Color changes in the heatmap represent changes in compound content, and the cluster analysis (Fig. 3C) showed that the colored areas of the five EVOOs were differentiated from one another, thus representing differences in the type and content of the metabolites.

### 3.6 Screening of phenolic compounds for potential bitterness and astringency

Bitterness and astringency of EVOO are related with cultivar and are important indicators of the quality of EVOO. The hydroxyl (–OH) and glycosidic bonds of the phenolic compounds bind to TAS2R on the tongue, triggering bitter sensory signals (Qiao et al., 2024). In addition, phenolic compounds and tannins cause astringency, and their types and concentrations determine their intensity (Huang & Xu, 2021). Studies have indicated that bitterness and astringency in EVOO were primarily derived from phenolic compounds and correlate with their type and content (Cliceri et al., 2021). Therefore, bitterness and astringency were selected from the nine taste sensations to further analyze their correlation with type and content of phenolic compounds in five EVOOs.

**Fig. 4:**
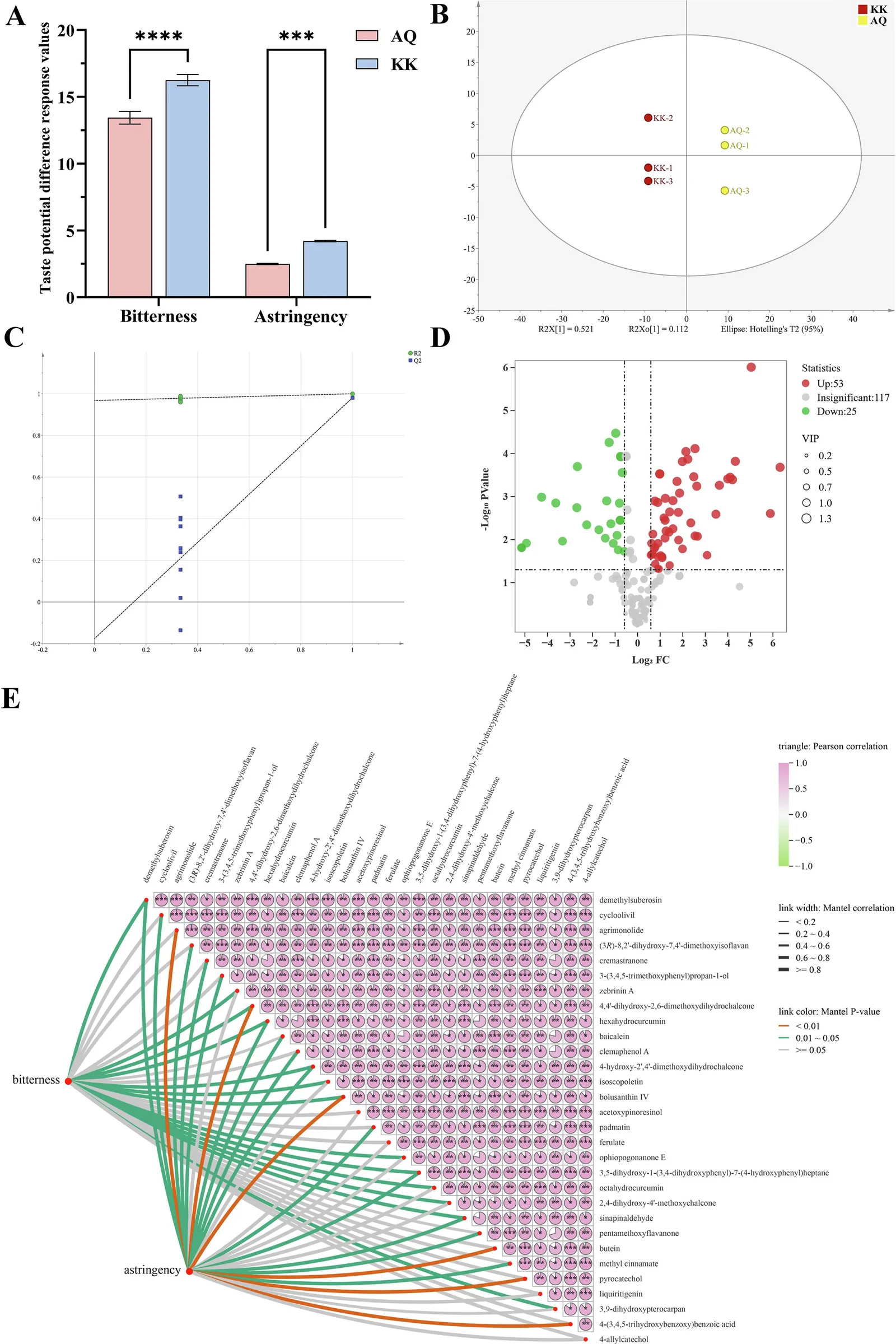
Screening of phenolic compounds with potential bitterness and astringency in EVOOs. (A) comparison of KK and AQ bitterness and astringency, ****p < 0.0001, ***p < 0.001; (B) OPLS-DA of KK and AQ; (C) 200 permutation tests for OPLS-DA; (D) volcano plots showing the differences in phenolic metabolites between KK and AQ. (E) Mantel test of bitterness and astringency.

Based on E-tongue taste analysis of five EVOOs, bitterness and astringency of KK were significantly different from AQ (Table 2 and Fig. 4A). These findings are in agreement to the research by Tura et al. (2013), who found that KK cultivated in Italy exhibited remarkably higher levels of bitterness and astringency compared to AQ. Consequently, to further examine the material basis underlying the significant differences in bitterness and astringency between KK and AQ, we performed a screen and a differential analysis of the phenolic metabolites in KK and AQ using comparative analyses.

The phenolic differential metabolites of AQ and KK, which may be the potential chemical components contributing to the bitterness and astringency, were further examined by OPLS-DA. Fig. 4B and C showed that OPLS-DA demonstrated high forecasting performance, with an R^2^X of 0.765 and a Q^2^ value of 0.999, which indicates a robust predictive ability without overfitting. KK and AQ were separated into two clusters, indicating significant differences between the components of the two samples. In addition, the OPLS-DA model was validated through 200 permutation tests, yielding R^2^ = 0.967 and Q^2^ = −0.176. This further indicated that the model was stable and credible. Based on the results of OPLS-DA, the values for the threshold variable important projection (VIP) and the *p*-value were calculated. A volcano plot was used to screen phenolic differential metabolites of KK and AQ with the parameters of VIP > 1 and *p* < 0.05 (Fig. 4D). A total of 78 phenolic differential metabolites from KK and AQ were selected, with 53 up-regulated and 25 down-regulated. These findings indicated that the greater bitterness and astringency of KK relative to AQ might be attributable to the 53 upregulated phenolic metabolites.

The criteria of VIP > 1, fold change (FC) >2 and *p* < 0.01 were used for screening differential phenolic metabolites (Liu et al., 2021). Thirty phenolic metabolites were screened (Table 3), containing phenolic acids (10), lignans (4), chalcones (4), coumarins (3), dihydroflavonoids (2), flavonoids (2), isoflavonoids (1), and other phenolic compounds (4). They may represent phenolic compounds that impart bitterness and astringency to the EVOOs. The chemical structures and contents are shown in Fig. S2.

**Table 3:** Phenolic differential metabolites of KK and AQ.

No	Classification	Compounds	VIP	FC	*p*
1	phenolic acids (10)	3-(3,4,5-trimethoxyphenyl)propan-1-ol	1.23	2.68	0.002269
2		hexahydrocurcumin	1.19	5.82	0.008196
3		ferulate	1.26	4.41	0.000090
4		3,5-dihydroxy-1-(3,4-dihydroxyphenyl)-7-(4-hydroxyphenyl)heptane	1.26	79.69	0.000208
5		octahydrocurcumin	1.25	3.36	0.000443
6		sinapinaldehyde	1.23	59.20	0.002471
7		methyl cinnamate	1.25	6.15	0.000572
8		pyrocatechol	1.25	5.62	0.000344
9		4-(3,4,5-trihydroxybenzoxy)benzoic acid	1.26	18.45	0.000403
10		4-allylcatechol	1.26	4.67	0.000134
11	lignans (4)	cycloolivil	1.26	20.16	0.000151
12		clemaphenol A	1.22	5.13	0.004076
13		bolusanthin IV	1.20	2.62	0.006711
14		acetoxypinoresinol	1.27	32.86	0.000001
15	chalcones (4)	4,4′-dihydroxy-2,6-dimethoxydihydrochalcone	1.25	2.96	0.001240
16		4-hydroxy-2′,4′-dimethoxydihydrochalcone	1.25	3.63	0.000829
17		butein	1.23	11.10	0.002557
18		2,4-dihydroxy-4′-methoxychalcone	1.23	3.49	0.002323
19	coumarins (3)	demethylsuberosin	1.26	3.96	0.000153
20		agrimonolide	1.26	17.21	0.000355
21		isoscopoletin	1.26	16.00	0.000386
22	dihydroflavonols (2)	padmatin	1.25	12.34	0.000546
23		liquiritigenin	1.24	2.34	0.001096
24	flavonoids (2)	baicalein	1.22	2.40	0.006294
25		pentamethoxyflavanone	1.20	6.29	0.008380
26	isoflavonoids (1)	3,9-dihydroxypterocarpan	1.19	2.32	0.009243
27	other flavonoids (4)	(3*R*)-8,2′-dihydroxy-7,4′-dimethoxyisoflavan	1.26	5.84	0.000077
28		cremastranone	1.23	2.33	0.003562
29		ophiopogonanone E	1.22	2.96	0.005575
30		zebrinin A	1.21	2.25	0.003135

The Mantel test is a powerful statistical tool to assess the correlation between distance matrices. It has been widely applied to multidimensional analyses of food flavor and taste (Hu et al., 2025; Ping et al., 2025). In the present study, correlation analyses were performed between the 30 phenolic compounds with bitterness and astringency in EVOO using Mantel test. The color and width of the lines connecting bitterness and astringency represent the statistical significance and the R statistic, respectively. The orange, green, and gray lines represent extremely remarkable (*p* < 0.01), remarkable (*p* < 0.05), and insignificant correlations (*p* > 0.05), respectively (Tang et al., 2025). As depicted shown Fig. 4E, bitterness was significantly associated with zebrinin A, isoscopoletin, ophiopogonanone E, octahydrocurcumin, 2,4-dihydroxy-4′-methoxychalcone, and 3,9-dihydroxypterocarpan. Astringency was significantly associated with cycloolivil, agrimonolide, (3*R*)-8,2′-dihydroxy-7,4′-dimethoxyisoflavan, cremastranone, 3-(3,4,5-trimethoxyphenyl)propan-1-ol, baicalein, clemaphenol A, padmatin, pentamethoxyflavanone, butein, methyl cinnamate, pyrocatechol, and 4-(3,4,5-trihydroxybenzoxy)benzoic acid. Moreover, demethylsuberosin, hexahydrocurcumin, 4,4′-dihydroxy-2,6-dimethoxydihydrochalcone, 4-hydroxy-2′,4′-dimethoxydihydrochalcone, bolusanthin IV, 3,5-dihydroxy-1-(3,4-dihydroxyphenyl)-7-(4-hydroxyphenyl)heptane, and sinapinaldehyde were concurrently significantly associated with bitterness and astringency. Overall, following electronic tongue and UPLC-MS/MS determinations, comparative analyses combined with Mantel test revealed that 26 key phenolic compounds identified in EVOOs exhibited significant correlations with bitterness and astringency. Among these, 6 and 13 phenolic compounds were significantly associated with bitterness and astringency, respectively, while 7 phenolic compounds were significantly associated with both bitterness and astringency. The identification of these compounds will contribute to an insight into origin of the bitterness and astringency taste of EVOO, and offer bases for exploitation of premium EVOO.

### 3.7 Verification of the bitterness of phenolic compounds based on bitter X and molecular docking

Studies have found that the perception of astringency originates in the trigeminal nerve, and its mechanism of action is more complex compared with that of bitterness (Canon et al., 2021), which makes it difficult to identify the underlying mechanism through molecular docking. Therefore, in the present study, we used molecular docking solely to speculate on the potential action mechanism of bitter substances. BitterX can predict binding capacity between small molecules and bitter taste receptors (TAS2R), thereby enabling bitterness agent validation and bitter taste receptor identification (Shi, Li et al., 2025). Herein, 13 bitter compounds were recognized based on BitterX and the Mantel test. BitterX predicted the strongest TAS2R receptor corresponding to the 13 compounds. Of these, 2,4-dihydroxy-4′-methoxychalcone, 4,4′-dihydroxy-2,6-dimethoxydihydrochalcone, 4-hydroxy-2′,4′-dimethoxydihydrochalcone, bolusanthin IV, demethylsuberosin, isoscopoletin, ophiopogonanone E, sinapinaldehyde, and zebrinin A shared the bitter taste receptor protein TAS2R14; whereas TAS2R39 is the bitter taste receptor protein associated with 3,5-dihydroxy-1-(3,4-dihydroxyphenyl)-7-(4-hydroxyphenyl) heptane, 3,9-dihydroxypterocarpan, hexahydrocurcumin, and octahydrocurcumin (Table S4).

**Fig. 5:**
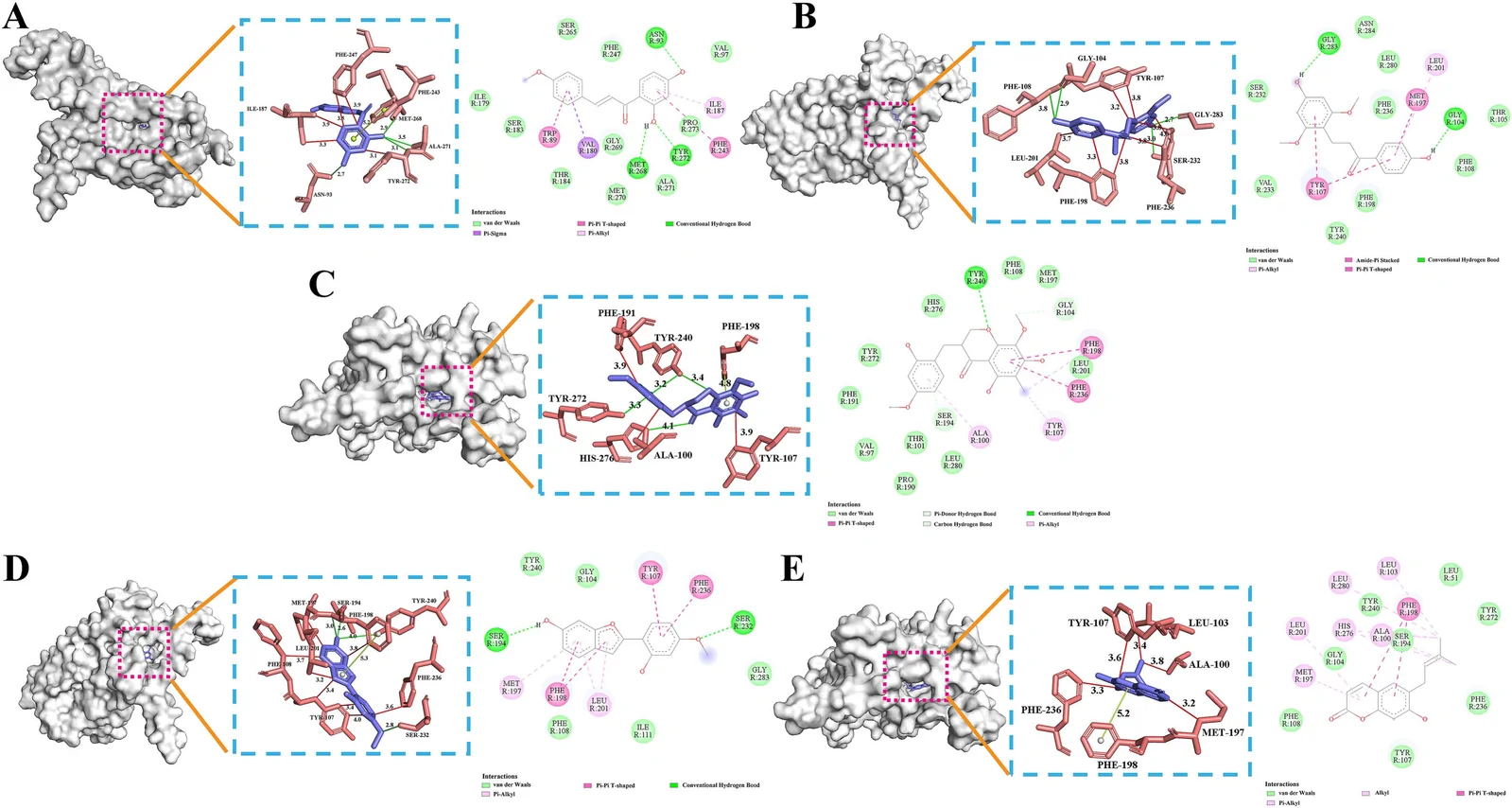
The results of a partial molecular docking. (A) 2,4-dihydroxy-4′-methoxychalcone with TAS2R14, (B) 4,4′-dihydroxy-2,6-dimethoxydihydrochalcone with TAS2R14, (C) ophiopogonanone E with TAS2R14, (D) bolusanthin IV with TAS2R14, and (E) demethylsuberosin with TAS2R14.

The binding score is a key indicator of the strength of intermolecular interactions. A lower value indicates a more stable binding between ligand and the receptor (Zhu et al., 2024). To further examine the potential mechanisms, 13 bitter compounds were molecularly docked with the bitter taste receptor (proteins, TAS2R14 and TAS2R39). Molecular docking analysis revealed that all 13 bitter compounds effectively bind to bitter taste receptors through hydrogen bonds and hydrophobic interactions, with binding scores ranging from −1.82 to −7.90 kcal/mol (Table S4). In particular, the results of 3D and 2D molecular docking between the top five compounds with strong binding affinity and bitter receptors are shown in Fig. 5. The interactions between the five compounds and proteins included hydrogen interaction, van der Waals, alkyl, pi–alkyl, pi–sigma, and pi–pi T-shaped. As depicted in Fig. 5C, ophiopogonanone E formed multiple hydrogen bonds with TYR240, TYR272, and HIS276, and multiple pi–pi T-shaped and pi–alkyl with PHE198, PHE236, TYR107, and ALA100, respectively. In addition, ophiopogonanone E also exhibited van der Waals interactions with the amino acids of TAS2R14, containing MET197, PHE108, HIS276, TYR272, PHE191, VAL97, THR101, PRO190, and LEU280, making it the strongest binder to TAS2R14. This binding likely contributes to the potent bitterness perception for ophiopogonanone E (Bai et al., 2023).

## 4 Conclusions

In this study, E-nose, GC-IMS, E-tongue and UPLC-MS/MS were used to evaluate flavor and taste among five EVOOs. By analyzing the flavors, W1W, W2W and W5S showed significantly higher response values than the other sensors in the E-nose. A total of 33 VOCs were characterized by GC-IMS, containing 8 esters, 7 aromatic hydrocarbons, 5 ketones, 4 alcohols, 2 aldehydes, 2 olefins, 1 ether and 1 acid. ROAV analysis revealed 16 VOCs that contribute to the sweet, grassy, and fruity profile of EVOOs, with esters significantly influencing the flavor profiles. Moreover, E-tongue analysis revealed that taste profiles in five EVOOs were relatively similar, and presented a profile of sweetness, bitterness and astringency. UPLC-MS/MS was employed for examining composition of phenolic compounds in five EVOOs. A total of 202 phenolic constituents were identified, which primarily included phenolic acids (94, 46.52%), lignans (39, 19.31%) and coumarins (14, 6.93%). Furthermore, by combining the results of E-tongue, KK, and AQ, large differences in bitterness and astringency were selected for comparative analysis using comparative analyses and Mantel test approach. The 26 putative phenolic compounds were selected that likely contribute to bitterness and astringency of EVOOs. Furthermore, screened bitter compounds were further validated through BitterX tools and molecular docking, which revealed that all compounds efficiently bind with bitter receptors via hydrogen bonding and hydrophobic interactions. These findings provide insight into the regulatory network of EVOO flavor and, which may taste related metabolites and further improvement of improve EVOO quality.

## CRediT authorship contribution statement

**Boxiao Wu:** Writing – original draft, Funding acquisition, Formal analysis, Data curation. **Churan Li:** Supervision, Investigation, Data curation. **Qing Hu:** Resources, Investigation. **Bin Lu:** Visualization, Resources, Investigation, Funding acquisition. **Changwei Cao:** Visualization, Supervision. **Fang Li:** Supervision, Investigation. **Huan Kan:** Project administration, Methodology, Funding acquisition, Conceptualization. **Yun Liu:** Writing – review & editing, Validation, Resources, Project administration, Conceptualization.

## Declaration of competing interest

The authors declare that they have no known competing financial interests or personal relationships that could have appeared to influence the work reported in this paper.

## Data Availability

Data will be made available on request.

## References

[bb0005] Alahlah N., Salhi A., Elyoussfi A., Amhamdi H., Ahari M.H. (2025). Charting a decade of olive oil authentication: A bibliometric survey of spectroscopy and chemometrics (2014**–**2024). Microchemical Journal.

[bb0010] Bai G.P., Pan Y., Zhang Y., Li Y., Wang J., Wang Y., Cao J. (2023). Research advances of molecular docking and molecular dynamic simulation in recognizing interaction between muscle proteins and exogenous additives. Food Chemistry.

[bb0015] Bengana M., Bakhouche A., Lozano-Sánchez J., Amir Y., Youyou A., Segura-Carretero A., Fernández-Gutiérrez A. (2013). Influence of olive ripeness on chemical properties and phenolic composition of Chemlal extra-virgin olive oil. Food Research International.

[bb0020] Cai X.M., Zhu K.X., Li W.L., Peng Y.Q., Yi Y.W., Qiao M.F., Fu Y. (2024). Characterization of flavor and taste profile of different radish (*Raphanus sativus* L.) varieties by headspace-gas chromatography-ion mobility spectrometry (GC/IMS) and E-nose/tongue. Food Chemistry: X.

[bb0025] Caño-Carrillo I., Gilbert-López B., Ruiz-Samblás C., Molina-Díaz A., García-Reyes J.F. (2024). Virgin olive oil authentication using mass spectrometry-based approaches: A review. TrAC Trends in Analytical Chemistry.

[bb0030] Canon F., Belloir C., Bourillot E., Brignot H., Briand L., Feron G., Neiers F. (2021). Perspectives on astringency sensation: An alternative hypothesis on the molecular origin of astringency. Journal of Agricultural and Food Chemistry.

[bb0035] Cecchi L., Migliorini M., Giambanelli E., Cane A., Zanoni B., Canuti V., Melani F. (2022). Is the volatile compounds profile a suitable tool for authentication of virgin olive oils (*Olea europaea* L.) according to cultivars? A study by using HS-SPME-GC-MS and chemometrics. Food Control.

[bb0040] Cecchi L., Migliorini M., Mulinacci N. (2021). Virgin olive oil volatile compounds: Composition, sensory characteristics, analytical approaches, quality control, and authentication. Journal of Agricultural and Food Chemistry.

[bb0045] Chen C., Sheng M.Q., Yuan H.B., Pan X., Tian H.X., Lou X.M. (2025). Perceptual interaction of aroma compounds and their effects on the enhancement of flavor quality in foods: A review. Trends in Food Science & Technology.

[bb0050] Chen L., Yin S.X., Dong S.Q., Xu P., Liu Y.L., Xiang X.L., Ye L. (2024). A new insight into the key matrix components for aftertaste in *Ampelopsis grossedentata* (vine tea) infusion: From the intensity and duration of taste profiles using non-targeted metabolomics and molecular simulation. Food Chemistry.

[bb0055] Chen X.M., Yang D.D., Huang L., Li M.Q., Gao J.H., Liu C., Xu R.C. (2024). Comparison and identification of aroma components in 21 kinds of frankincense with variety and region based on the odor intensity characteristic spectrum constructed by HS–SPME–GC–MS combined with E-nose. Food Research International.

[bb0060] Cliceri D., Aprea E., Menghi L., Endrizzi I., Gasperi F. (2021). Variability in the temporal perception of polyphenol-related sensations in extra virgin olive oil and impact on flavor perception. Food Quality and Preference.

[bb0065] Damiani T., Cavanna D., Serani A., Dall'Asta C., Suman M. (2020). GC-IMS and FGC-Enose fingerprint as screening tools for revealing extra virgin olive oil blending with soft-refined olive oils: A feasibility study. Microchemical Journal.

[bb0070] Dana L.M., Chapman K., Dixon H., Miller C., Neal B., Kelly B., Pettigrew S. (2021). The relative importance of primary food choice factors among different consumer groups: A latent profile analysis. Food Quality and Preference.

[bb0075] Díaz-Montaña E.J., Brignot H., Aparicio-Ruiz R., Thomas-Danguin T., Morales M.T. (2024). Phenols and saliva effect on virgin olive oil aroma release: A chemical and sensory approach. Food Chemistry.

[bb0080] Frankel E.N. (2011). Nutritional and biological properties of extra virgin olive oil. Journal of Agricultural and Food Chemistry.

[bb0085] Genovese A., Caporaso N., Sacchi R. (2021). Flavor chemistry of virgin olive oil: An overview. Applied Sciences.

[bb0090] Harzalli U., Rodrigues N., Veloso A.C., Dias L.G., Pereira J.A., Oueslati S., Peres A.M. (2018). A taste sensor device for unmasking admixing of rancid or winey-vinegary olive oil to extra virgin olive oil. Computers and Electronics in Agriculture.

[bb0095] Hu Y.Y., Liao J.L., Qian W.Z., Fan S.J., Xiao X.Y., Yang Y., Gao S. (2025). Metabolomics, E-tongue and HS-SPME-GC–MS reveal the smoking process of *Prunus mume*: Changes in flavor and chemical compositions. Food Chemistry.

[bb0100] Huang R., Xu C.M. (2021). An overview of the perception and mitigation of astringency associated with phenolic compounds. Comprehensive Reviews in Food Science and Food Safety.

[bb0105] Kalua C.M., Allen M.S., Bedgood D.R., Bishop A.G., Prenzler P.D., Robards K. (2007). Olive oil volatile compounds, flavour development and quality: A critical review. Food Chemistry.

[bb0110] Kanwal N., Musharraf S.G. (2024). Analytical approaches for the determination of adulterated animal fats and vegetable oils in food and non-food samples. Food Chemistry.

[bb0115] Li Y., Blank I., Liu X., Feng X.X., Fan Y.X., Yu Y.Y., Liu Y. (2025). Multi-omics elucidation of key odor-active compounds and their association with lipid-derived flavor precursors in textured peanut protein-based fermented sausage analogs. LWT - Food Science and Technology.

[bb0120] Liu K.Y., Wang L.Y., Jiang B., An J.S., Nian B., Wang D.P., Zhao M. (2021). Effect of inoculation with *Penicillium chrysogenum* on chemical components and fungal communities in fermentation of Pu-erh tea. Food Research International.

[bb0125] Liu Z.S., Wang H., Zhang J., Chen Q., He W., Zhang Y., Wang X.R. (2024). Comparative metabolomics profiling highlights unique color variation and bitter taste formation of Chinese cherry fruits. Food Chemistry.

[bb0130] Lü X.F., Ma J.Y., Yan H.Q., Yang L.H., Ren X.X., Guo J.W., Deng Y. (2021). Effect of degree of ripening on the quality of virgin olive oils produced in Longnan, China. Journal of the American Oil Chemists’ Society.

[bb0135] Moreira G.C., Carneiro C.N., Dos Anjos G.L., da Silva F., Santos J.L., Dias F.D.S. (2022). Support vector machine and PCA for the exploratory analysis of *Salvia officinalis* samples treated with growth regulators based in the agronomic parameters and multielement composition. Food Chemistry.

[bb0140] Ongalbek D., Tokul-Ölmez Ö., Şahin B., Küçükaydın S., Aydoğmuş-Öztürk F., Sıcak Y., Öztürk M. (2024). Classification of buckwheat honey produced in Kazakhstan according to their biochemical ingredients and bioactivities by chemometric approach. Food Chemistry.

[bb0145] Ortiz-Arrabal O., Bermejo-Casares F., Garzón I., Mesa-García M.D., Gómez-Llorente C., Alaminos M. (2023). Optimization of human skin keratinocyte culture protocols using bioactive molecules derived from olive oil. Biomedicine & Pharmacotherapy.

[bb0150] Ping C.Y., Deng X.Q., Guo Z.Y., Luo W., Li X., Xin S.L. (2024). Characterizing the flavor profiles of Linjiangsi broad bean (*Vicia faba* L.) paste using bionic sensory and multivariate statistics analyses based on ripening time and fermentation environment. Food Chemistry: X.

[bb0155] Ping C.Y., Li B., Gao Y.Y., Li X., Wang F. (2025). Exploring the mechanisms of flavor formation and polyphenolic changes in hop-infused sourdough bread affected by hop varieties and soaking methods. Food Chemistry: X.

[bb0160] Qiao K.N., Zhao M.X., Huang Y., Liang L., Zhang Y.Y. (2024). Bitter perception and effects of foods rich in bitter compounds on human health: A comprehensive review. Foods.

[bb0165] Ríos-Reina R., Aparicio-Ruiz R., Morales M.T., García-González D.L. (2023). Contribution of specific volatile markers to green and ripe fruity attributes in extra virgin olive oils studied with three analytical methods. Food Chemistry.

[bb0170] Rodrigues N., Marx Í.M., Casal S., Dias L.G., Veloso A.C., Pereira J.A., Peres A.M. (2019). Application of an electronic tongue as a single-run tool for olive oils’ physicochemical and sensory simultaneous assessment. Talanta.

[bb0175] Shi X.L., Xiao Y.L., Zhao N.Y., Yao L., Li H.Y., Huang J.H., Wang X.G. (2025). Bitter and astringent characteristics of alkalized cocoa powder: Analysis using UPLC-Q-TOF-MS/MS and molecular simulation. Food Chemistry.

[bb0180] Shi X.Y., Li Y., Huang D.J., Chen S.W., Zhu S. (2025). Characterization and discrimination of volatile compounds in roasted arabica coffee beans from different origins by combining GC-TOFMS, GC-IMS, and GC-E-nose. Food Chemistry.

[bb0185] Spangenberg J.E., Lantos I. (2024). Fingerprinting of monovarietal olive oils from Argentina and Uruguay via stable isotope, fatty acid profile, and chemometric analyses. Food Chemistry.

[bb0190] Su C.J., Sun J.F., Zhu W.Z., Peng L. (2018). History, distribution, and potential of the olive industry in China: A review. Sustainability.

[bb0195] Sung J., Frost S., Suh J.H. (2025). Progress in flavor research in food: Flavor chemistry in food quality, safety, and sensory properties. Food Chemistry: X.

[bb0200] Tang G.Y., Huang Y., Zhang T.T., Wang Q.Q., Crommen J., Fillet M., Jiang Z.J. (2018). Determination of phenolic acids in extra virgin olive oil using supercritical fluid chromatography coupled with single quadrupole mass spectrometry. Journal of Pharmaceutical and Biomedical Analysis.

[bb0205] Tang J., Lin B., Shan Y.M., Zhang G., Zhu L.P., Jiang W., Du H. (2025). Application of indigenous *Saccharomycopsis fibuligera* for light-flavor baijiu fermentation: Changes of microbial community and flavor metabolism. Current Research in Food Science.

[bb0210] Tomé-Rodríguez S., Ledesma-Escobar C.A., Penco-Valenzuela J.M., Priego-Capote F. (2021). Cultivar influence on the volatile components of olive oil formed in the lipoxygenase pathway. LWT - Food Science and Technology.

[bb0215] Tura D., Failla O., Bassi D., Attilio C., Serraiocco A. (2013). Regional and cultivar comparison of Italian single cultivar olive oils according to flavor profiling. European Journal of Lipid Science and Technology.

[bb0220] Wang D.Q., Wang J., Lang Y., Huang M.Q., Hu S.L., Liu H.Q., Dong W. (2025). Interactions between food matrices and odorants: A review. Food Chemistry.

[bb0225] Wang Y.Y., Ma Y.H., Duan J.L., Wang B., Ma T.Z., Jiang Y.M., Zhang B. (2025). Discrimination and characterization of the volatile organic compounds in red and black raspberry wines fermented with different commercial *Saccharomyces cerevisiae*: An integrated analysis using E-nose, GC-MS, GC-IMS, and multivariate statistical models. Food Chemistry.

[bb0230] Xie X., Wang Y.H., Wen B.S., Tian J.L., Cheng Z., Tang S.Y., Li B. (2025). Characterization and metabolism pathway of volatile compounds in blueberries of different varieties and origins analyzed *via* HS-GC-IMS and HS-SPME-GC–MS. Food Chemistry.

[bb0235] Zhang B.H., Wang Y., Wang J.P., Zhang Y.M., Wang W., Cao J.X., Teng W. (2025). The establishment of ham grade, sensory scores and key flavor substances prediction models for Jinhua ham via E-nose combined with machine learning. Food Chemistry.

[bb0240] Zhang K., Ma Q.L., Wang Y., Yuan Z.C., Yang Z.W., Luo X., Deng Q. (2024). Transcriptome and biochemical analyses reveal phenolic compounds-mediated flavor differences in loquat (*Eriobotrya japonica* Lindl.) cultivars Chunhua no. 1 and Dawuxing. Food Chemistry: X.

[bb0245] Zhu L.J., Pan Y.Y., Li Y.Y., Zhou Y.J., Liu H., Liu X.Y. (2024). Molecular mechanisms of bitterness and astringency in the oral cavity induced by soyasaponin. Food Science and Human Wellness.

