# Supplementary material for: Characterization and discrimination of flavor and taste profiles in olive oil from different varieties using E-nose, GC-IMS, E-tongue, and UPLC-MS/MS
Source: Food Chem X. 2026 Aug 25;38:104354. doi: 10.1016/j.fochx.2026.104354 (PMC13543986; doi:10.1016/j.fochx.2026.104354)
Supplement: Supplementary file 1 [file mmc1.docx]

**Supplementary material**

**Characterization and discrimination of** **flavor** **and taste profiles in olive oil from different** **varieties using** **E-nose,** **GC-IMS, E-tongue, and** **UPLC-MS/MS**

Boxiao Wu ^a,b^, Churan Li ^b^, Qing Hu ^b^, Bin Lu ^b,**^, Changwei Cao ^a^, Fang Li ^a^, Huan Kan ^a^, Yun Liu ^a,*^

^a^ *Forest Resources Exploitation and Utilization Engineering Research Center for Grand Health of Yunnan Provincial Universities, Southwest Forestry University, Kunming 650224, China*

^b^ *Yunnan Technology Innovation Center of Woody Oil,* *Yunnan Academy of Forestry and Grassland, Kunming 650204, China*

^*^ Corresponding author at: Forest Resources Exploitation and Utilization Engineering Research Center for Grand Health of Yunnan Provincial Universities, Southwest Forestry University, Kunming 650224, China

^**^ Corresponding author at: Yunnan Technology Innovation Center of Woody Oil, Yunnan Academy of Forestry and Grassland, Kunming 650204, China

*E-mail addresses*: kmlubin@163.com (B. Lu), liuyun@swfu.edu.cn (Y. Liu).

**Figures:**

**Fig. S1.** Characterization and analysis of taste in five EVOOs by E-tongue. (A) Radar chart; (B) PCA.

**Fig. S2.** The content and structural of potential bitterness and astringency presenting phenolic in EVOOs. *****p* < 0.0001, ****p* < 0.001, ***p* < 0.01.

**Tables:**

**Table S1.** The description of E-nose sensor performance.

| NO. | Sensor | Sensitivity |
| --- | --- | --- |
| 1 | W1C | Sensitive to aromatics, benzene |
| 2 | W3C | Sensitive to ammonia, aromatic components |
| 3 | W5C | Sensitive to alkane aromatic components |
| 4 | W1S | Sensitive to short-chain alkanes, methane |
| 5 | W2S | Sensitive to alcohol, ether, aldehydes, and ketones |
| 6 | W3S | Sensitive to long-chain alkanes |
| 7 | W5S | Sensitive to nitrogen oxides |
| 8 | W6S | Sensitive to hydrogen |
| 9 | W1W | Sensitive to inorganic sulfides |
| 10 | W2W | Sensitive to aromatic components, organic sulfides |

**Table S2.** The description of E-tongue sensor performance.

| NO. | Sensor | Taste | Taste information | |
| --- | --- | --- | --- | --- |
|  |  |  | Pre-taste | After-taste |
| 1 | C00 | Bitterness | Bitterness | Aftertaste-bitterness |
| 2 | AE1 | Astringency | Astringency | Aftertaste-astringency |
| 3 | CA0 | Sourness | Sourness | – |
| 4 | CT0 | Saltness | Saltness | – |
| 5 | AAE | Umami | Umami | Richness |
| 6 | GL1 | Sweetness | Sweetness | – |

**Table S3.** VOCs of different EVOOs varieties using GC-IMS.

| Classification | Compound | Peak area | | | | |
| --- | --- | --- | --- | --- | --- | --- |
|  |  | AQ | EP | PC | FO | KK |
| Esters  (8) | phenethyl acetate | 2789.01 ± 362.69^a^ | 2371.33 ± 249.62^ab^ | 2211.94 ± 249.90^b^ | 2630.07 ± 32.86^ab^ | 2718.57 ± 42.48^a^ |
|  | (*E*)-ethyl crotonate | 494.52 ± 12.35^a^ | 436.57 ± 89.25^a^ | 453.35 ± 132.28^a^ | 442.32 ± 89.08^a^ | 237.33 ± 29.99^b^ |
|  | ethyl isobutyrate | 1313.42 ± 39.31^a^ | 59.75 ± 2.53^b^ | 34.47 ± 4.55^bc^ | 33.83 ± 2.61^bc^ | 27.58 ± 0.76^c^ |
|  | ethyl acetate | 5726.66 ± 71.29^a^ | 567.77 ± 34.76^b^ | 5509.97 ±674.90^a^ | 5258.66 ± 26.91^a^ | 5384.53 ± 56.33^a^ |
|  | ethyl butanoate | 120.25 ± 14.15^b^ | 714.21 ± 32.49^a^ | 62.24 ± 13.15^d^ | 86.94 ± 5.27^cd^ | 91.89 ± 4.96^bc^ |
|  | ethyl isovalerate | 207.24 ± 7.33^a^ | 99.95 ± 13.39^c^ | 172.42 ± 37.14^b^ | 220.23 ± 6.10^a^ | 171.93 ± 8.87^b^ |
|  | methyl-2-methyl butanoate | 12.43 ± 0.85^d^ | 44.79 ± 2.85^c^ | 58.40 ± 10.96^b^ | 91.29 ± 2.12^a^ | 96.88 ± 7.20^a^ |
|  | ethyl propionate | 3304.10 ± 42.20^a^ | 59.64 ± 2.10^b^ | 35.71 ± 8.31^b^ | 36.50 ± 10.18^b^ | 45.84 ± 5.09^b^ |
| Aromatic Hydrocarbon  (7) | meta-cresol | 1992.88 ± 435.31^a^ | 964.92 ± 315.65^b^ | 322.90 ± 27.82^c^ | 392.73 ± 49.06^c^ | 563.29 ± 71.46^bc^ |
|  | methyl salicylate | 2286.28 ± 533.92^a^ | 994.93 ±414.23^bc^ | 862.65 ± 275.77^c^ | 1350.36 ± 51.17^bc^ | 1557.28 ± 271.14^b^ |
|  | 2-acetyl-3-methylpyrazine | 1104.56 ± 65.95^a^ | 156.63 ± 25.59^e^ | 537.34 ± 59.39^b^ | 242.85 ± 20.43^d^ | 385.14 ± 9.77^c^ |
|  | ethylpyrazine | 9433.99 ± 218.58^b^ | 14120.40 ± 209.62^a^ | 3856.04 ± 411.34^d^ | 3846.60 ± 165.37^d^ | 5280.47 ± 175.07^c^ |
|  | 2,3-dimethyl-5-ethylpyrazine | 372.17 ± 9.30^b^ | 410.41 ± 10.41^b^ | 550.79 ± 46.77^a^ | 73.55 ± 21.46^c^ | 534.14 ± 31.04^a^ |
|  | 2,3-dimethylpyrazine | 759.21 ± 112.66^ab^ | 439.33 ± 9.88^c^ | 659.14 ± 41.69^b^ | 658.41 ± 38.73^b^ | 830.48 ± 22.52^a^ |
|  | pyrazine | 248.96 ± 10.12^c^ | 853.45 ± 48.50^b^ | 1066.79 ± 14.17^a^ | 1098.38 ± 75.51^a^ | 1066.94 ± 20.22^a^ |
| Ketones  (5) | 2-octanone | 2937.91 ± 350.87^a^ | 186.58 ± 178.31^b^ | 141.06 ± 47.57^b^ | 91.03 ± 8.06^b^ | 90.00 ± 3.73^b^ |
|  | 4,5-dihydro-3(2*H*)-thiophenone | 2748.26 ± 69.56^b^ | 3107.74 ± 229.67^a^ | 1489.50 ± 84.61^c^ | 1396.82 ± 48.23^c^ | 1586.92 ± 26.11^c^ |
|  | 2-methyloxolan-3-one | 385.78 ± 9.56^a^ | 35.32 ±3.38^b^ | 31.60 ± 3.34^b^ | 22.70 ± 2.34^c^ | 30.52 ± 4.38^bc^ |
|  | 3-hexanone | 238.36 ± 11.77^c^ | 293.44 ± 24.73^b^ | 343.34 ± 44.74^a^ | 297.98 ± 20.59^ab^ | 240.23 ± 8.47^c^ |
|  | 2,3-pentanedione | 1260.04 ± 22.45^c^ | 1551.22 ± 26.89^b^ | 1932.40 ± 276.08^a^ | 1960.83 ± 63.87^a^ | 2079.03 ± 57.93^a^ |
| Alcohols  (4) | 2,3-butanediol | 1644.55 ± 57.02^a^ | 569.66 ± 20.81^c^ | 1591.59 ± 290.49^a^ | 1259.54 ± 128.69^b^ | 1406.65 ± 39.77^ab^ |
|  | isopentyl alcohol | 572.30 ± 12.38^a^ | 137.12 ± 44.29^d^ | 354.25 ± 99.30^b^ | 250.61 ± 23.01^c^ | 281.24 ± 10.12^bc^ |
|  | 1-penten-3-ol | 4092.15 ± 47.77^b^ | 4083.84 ± 341.24^b^ | 6754.31 ± 549.90^a^ | 1861.42 ± 65.52^d^ | 3047.16 ± 97.43^c^ |
|  | 2-butanol | 4694.16 ± 92.16^ab^ | 3763.28 ± 391.26^c^ | 4270.33 ± 338.83^b^ | 4517.92 ± 59.30^ab^ | 4764.12 ± 92.37^a^ |
| Aldehydes  (2) | (*E*)-2-octenal | 2237.05 ± 111.18^a^ | 893.23 ± 92.17^e^ | 1559.08 ± 38.69^b^ | 1026.93 ± 25.99^d^ | 1296.17 ± 17.20^c^ |
|  | 3-methyl-2-butenal | 491.58 ± 27.92^c^ | 322.53 ± 26.34^c^ | 1274.14 ± 372.11^a^ | 900.63 ± 187.91^b^ | 899.88 ± 144.36^b^ |
| Olefins  (2) | 1-octene | 539.55 ± 8.26^a^ | 367.98 ± 21.92^c^ | 438.56 ± 86.52^bc^ | 489.25 ± 14.78^ab^ | 484.61 ± 11.83^ab^ |
|  | 2,4-dimethyl-1-heptene | 89.59 ± 6.77^b^ | 92.67 ± 1.94^b^ | 102.15 ± 14.17^b^ | 145.70 ± 17.30^a^ | 94.56 ± 10.27^b^ |
| Ethers (1) | rose oxide | 1267.68 ± 21.00^a^ | 132.62 ± 35.58^b^ | 147.09 ± 2.10^b^ | 123.74 ± 1.15^b^ | 116.34 ± 3.13^b^ |
| Acids (1) | pentanoic acid | 1020.69 ± 18.45^a^ | 1060.75 ± 11.73^a^ | 1066.56 ± 226.27^a^ | 1213.70 ± 21.95^a^ | 1029.51 ± 18.44^a^ |

Significant differences between groups are indicated by different letters in the same row of data (*p* < 0.05).

**Table S4.** Molecular docking score of the selected compounds on TAS2R14 (UniProtKB ID: Q9NYV8) or TAS2R39 (UniProtKB ID: P59534).

| NO. | Sensor | Binding score (kcal/mol) | |
| --- | --- | --- | --- |
|  |  | TAS2R14 | TAS2R39 |
| 1 | 2,4-dihydroxy-4'-methoxychalcone | –7.50 | – |
| 2 | 4,4'-dihydroxy-2,6-dimethoxydihydrochalcone | –6.77 | – |
| 3 | 4-hydroxy-2',4'-dimethoxydihydrochalcone | –6.58 | – |
| 4 | bolusanthin IV | –6.8 | – |
| 5 | demethylsuberosin | –6.78 | – |
| 6 | isoscopoletin | –5.67 | – |
| 7 | ophiopogonanone E | –7.90 | – |
| 8 | sinapinaldehyde | –5.96 | – |
| 9 | zebrinin A | –6.73 | – |
| 10 | 3,5-dihydroxy-1-(3,4-dihydroxyphenyl)-7-(4-hydroxyphenyl)heptane | – | –2.59 |
| 11 | 3,9-dihydroxypterocarpan | – | –6.17 |
| 12 | hexahydrocurcumin | – | –3.38 |
| 13 | octahydrocurcumin | – | –1.82 |
